# Numerical Response Surfaces of Volume of Ablation and Retropulsion Amplitude by Settings of Ho:YAG Laser Lithotripter

**DOI:** 10.1155/2018/8261801

**Published:** 2018-03-07

**Authors:** Jian J. Zhang, Jonathan Rutherford, Metasebya Solomon, Brian Cheng, Jason R. Xuan, Jason Gong, Honggang Yu, Michael L. D. Xia, Xirong Yang, Thomas Hasenberg, Sean Curran

**Affiliations:** ^1^Boston Scientific Corporation, 3070 Orchard Drive, San Jose, CA 95134, USA; ^2^Boston Scientific Corporation, 300 Boston Scientific Way, Marlborough, MA 01752, USA

## Abstract

**Objectives:**

Although laser lithotripsy is now the preferred treatment option for urolithiasis due to shorter operation time and a better stone-free rate, the optimal laser settings for URS (ureteroscopic lithotripsy) for less operation time remain unclear. The aim of this study was to look for quantitative responses of calculus ablation and retropulsion by performing operator-independent experiments to determine the best fit versus the pulse energy, pulse width, and the number of pulses.

**Methods:**

A lab-built Ho:YAG laser was used as the laser pulse source, with a pulse energy from 0.2 J up to 3.0 J and a pulse width of 150 *μ*s up to 1000 *μ*s. The retropulsion was monitored using a high-speed camera, and the laser-induced craters were evaluated with a 3-D digital microscope. The best fit to the experimental data is done by a design of experiment software.

**Results:**

The numerical formulas for the response surfaces of ablation speed and retropulsion amplitude are generated.

**Conclusions:**

The longer the pulse, the less the ablation or retropulsion, while the longer pulse makes the ablation decrease faster than the retropulsion. The best quadratic fit of the response surface for the volume of ablation varied nonlinearly with pulse duration and pulse number.

## 1. Introduction

Calculi occur in the urinary tract (kidney, ureter, bladder, and urethra) affecting about 10% of the population with a high recurrence rate of ~50% [1–4]. Urinary calculi are crystalline deposits, also known as the kidney/ureter/bladder/urethra calculus or uroliths, which occur in the urinary system. The condition causes the patient severe acute discomfort and pain. Based on the number of patients, urinary calculus disease is the 3rd largest area in urology after urinary tract infection and prostate disease. Shockwave lithotripsy (SWL) and ureteroscopic laser lithotripsy (URS) are the most commonly performed procedures in the United States for the treatment of patients with urinary calculi [5, 6]. The retrospective study in [7] revealed superior stone-free rate (SFR) results for renal stones < 1.5 cm for URS compared with SWL.

Intracorporeal laser lithotripsy for fragmentation of urinary calculi began in the mid-1980s [8–13]. The clinically available laser lithotrites are the pulsed-dye laser, the frequency-doubled pulsed Nd:YAG laser (FREDDY), and the Ho:YAG laser [14–16]. And comparing to nanosecond Nd:YAG lasers, the long-pulse Ho:YAG laser is the most efficient and versatile tool for lithotripsy among all the commercially available lasers. The Ho:YAG laser can fragment all calculus compositions and produces less calculus migration (retropulsion) during treatment than the short-pulsed lasers [17–21]. Since, shortly after its introduction in the 1990s, the Ho:YAG laser has been the favored lithotrite for the treatment of urinary calculus. It is a solid-state pulsed-wave laser operating at a wavelength of 2.13 *μ*m. This wavelength is readily absorbed by water (~26 cm^−1^ [22]), making it ideal for lithotripsy in the urinary tract by imparting a broad margin of safety [23–25]. Calculus is fragmented using a process of laser ablation, whereby a vaporization bubble forms and surrounds the fiber tip, which then destabilizes the calculus [26]. The energy is delivered through flexible silica fibers, allowing them to be passed through the working channel of all endoscopes commonly used in urology. The Ho:YAG is capable of fragmenting calculus of all known composition, including hard calcium oxalate monohydrate, brushite, and cystine calculus [21, 23, 24, 26]. Aside from treating calculi, it can be used for soft tissue applications such as treating urinary strictures and ablating urothelial tumors. The high-powered variant can also be used for holmium laser enucleation of the prostate (HoLEP).

The dominant mechanism in Ho:YAG laser lithotripsy is photothermal along with minor effects of acoustic emission [25]. Direct light absorption of the urinary calculi increases the temperature of the irradiated volume above the ablation threshold, consequently causing the ejection of fragmented breakdown products. Besides, absorption of laser energy by water between calculus and fiber tip induces vapor bubble formation and collapse with shock wave generation. During laser-calculus interaction, the urinary calculus is subject to retropulsion forces caused by the combined effects of ablated particle ejection, interstitial water vaporization, and bubble expansion/collapse [27–29]. Thus, due to the recoil momentum, the calculus is displaced away from the light delivery fiber. Retropulsive calculus movement prolongs operation time due to the cumbersome process required to reorient the endoscopic fiber toward the new calculus position. Previous retropulsion studies quantified calculus retropulsion distance by varying optical pulse energy, pulse repetition rate, and fiber diameter [30–32]. Retropulsion increased with applied pulse energy and optical fiber diameter. Further, a recent study reported that a longer pulse width reduced calculus retropulsion distance during a procedure without diminishing ablation efficiency significantly [33].

Although laser lithotripsy is now the preferred treatment option for urolithiasis, and the rising prevalence of calculus disease in the United States has led to similarly increasing efforts to optimize ureteroscopic treatment [34–41], the optimal laser settings for URS for effective treatment remain unclear. This may be due in part to the flexibility operators currently adjusting holmium laser lithotripter parameters: pulse energy, pulse width, and pulse frequency. Understanding these adjustable parameters allows the urologist to enhance their control during laser lithotripsy procedures [42]. The aim of this study was to look for precise quantitative responses of calculus ablation and retropulsion by performing operator-independent, reproducible experiments to determine the best fit of surface responses on volume of ablation and retropulsion amplitude versus the pulse energy, pulse width, and the number of pulses. More detailed investigation of the optimal conditions for the ablation of other kinds of calculus samples and the fiber size/burn back effects will be conducted as a future study.

## 2. Materials and Methodology

### 2.1. Fiber

This study used a SureFlex™ Fiber, model S-LLF365, 365 *μ*m core diameter fiber (S-LLF365 SureFlex Fiber, Boston Scientific Corp., San Jose, California, USA).

### 2.2. Calculus Phantom

**Figure 1:**
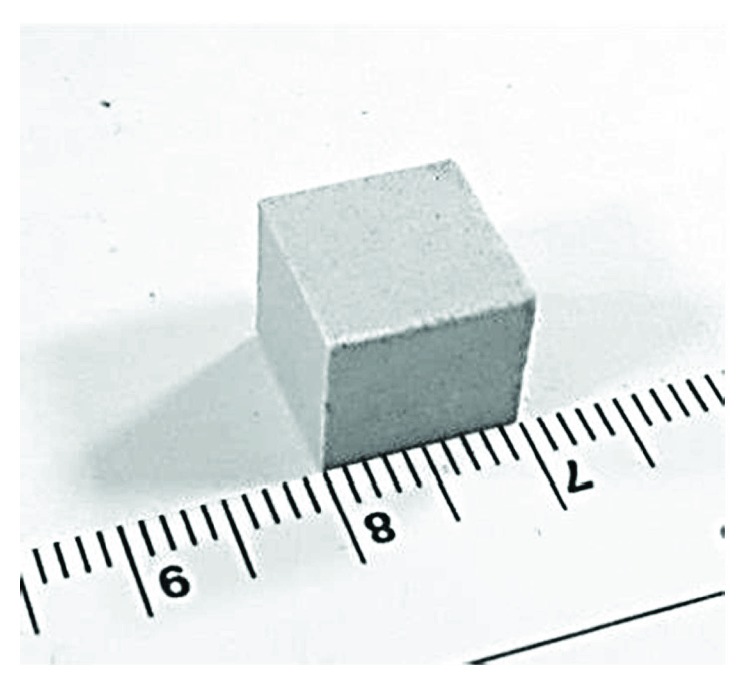
10 × 10 × 10 mm^3^ calculus phantom.

Calculus phantoms made of white gypsum cement used as tissue phantom for human kidney calculus (UtralCal®30, United States Gypsum Company, Chicago, IL) were widely used for laser lithotripsy studies by other researchers [43]. The tissue phantoms are prepared by mixing gypsum cement (500 g) with distilled water (0.23 liter) and allowing curing for at least 3 hours (overnight curing preferred). The cement was molded to have a size of 10 × 10 × 10 mm^3^ as shown in Figure 1. A cubic shape of the stone phantom was chosen because of ease of construction and its simple shape for controlled damage/retropulsion studies. The calculus phantom has an average mass of 1.8 g, and its tensile strength of 2 MPa, which is comparable with a tensile strength of human struvite (0.1 to 3.4 MPa) [44].

### 2.3. Laser System

**Figure 2:**
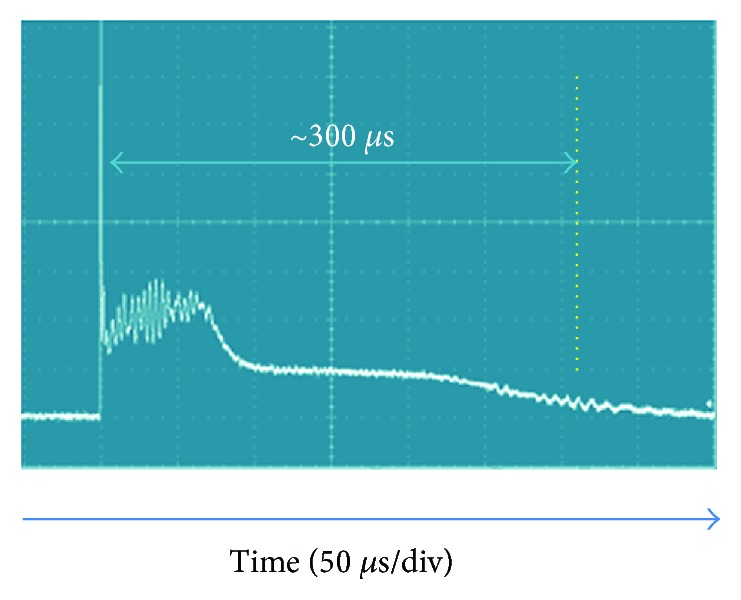
Optical pulse measured by a photodiode.

A custom pulsed Ho:YAG laser at 2.13 *μ*m, with a pulse energy from 0.2 J up to 3.0 J and a pulse width from 150 *μ*s up to 1000 *μ*s, was used as the laser pulse source.  Figure 2 shows a temporal pulse structure diagram of the Ho:YAG laser with a pulse duration (*τ*_p_) of ~300 *μ*s (from laser starting to ~10% of the middle plateau). This range of pulse duration is known to generate photo thermal effect to fragment the calculus [45].

### 2.4. Experimental Method and Setup

In this study, a lab-built Ho:YAG laser was used as the laser pulse source, with a pulse energy from 0.2 J up to 3.0 J and electrical pump pulse width from 150 *μ*s up to 1000 *μ*s with two fixtures designed to mimic the technique of calculus ablation and retropulsion. This lab-built laser is operated at 10 Hz throughout the test, and it can be programmed to emit a predefined number of pulses (from 1 to 999,999) with preheating pulse train (typically 30 pulses with 30 Hz and 100 *μ*s pulse width at operating voltage). A design of experiment software (Design-Expert 10, Minneapolis, MN, USA) is used in this study for the best fit of surface responses. This can not only cut down the number of test points but also generate a formula for the response surfaces of ablation speed and retropulsion amplitude. Plaster of Paris calculus phantoms were ablated at different energy levels (0.2, 0.5, 1, 2, and 3 J) and with a different number of pulses (1, 3, and 10) using different electrical pump pulse widths (333, 667, and 1000 *μ*s). The dynamics of the recoil action of a calculus phantom was monitored using a high-speed camera with a frame rate up to 1 million frames per second (Photron Fastcam SA5), and the laser-induced craters were evaluated with a 3-D digital microscope (Keyence VHX-900F).

**Figure 3:**
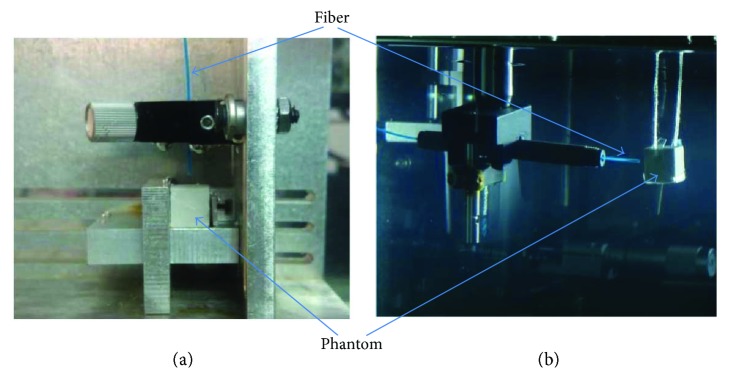
The pictures of the test setup, (a) ablation test setup with 10 mm phantom and (b) retropulsion test setup with 10 mm phantom.

Figure 3 are the pictures of the test setup, (a) ablation test setup and (b) retropulsion test setup. From the ablation test setup (a), the fiber (a 365 *μ*m core diameter fiber, S-LLF365 SureFlex Fiber, Boston Scientific Corporation, San Jose, CA, USA, delivers the laser pulse) was held vertically by a clamp, and its tip was in contact with the calculus phantom situated in a holder under the fiber. The stone was held fixed and immobile during the ablation study; this is to test the laser pulse ablation in a well-controlled setup without the stone movement. The whole setup was submerged in the distilled water. After the laser pulse and calculus interaction, the laser ablation crater volume in the phantom was measured by a digital microscope (VHX-900F, Keyence, Elmwood Park, NJ, USA). For the retropulsion test setup (b), a 365 *μ*m core diameter fiber (S-LLF365 SureFlex Fiber, Boston Scientific Corp., San Jose, California, USA) was held horizontally to deliver the laser pulse to the calculus phantom. An underwater pendulum is employed for retropulsion study, which consists of a calculus phantom cube with a dimension of 10 × 10 × 10 mm^3^. The calculus phantom is suspended in water by a sewing thread of ~200 mm in length. In order to control the rotational motion of the calculus in case the laser pulse from the fiber is not exactly pointed at the center of mass of the calculus phantom, the calculus was held in a clear plastic basket and 2 strings with a separation of ~10 mm are used to hang the phantom as shown in Figure 3(b). Since water has a relatively low viscosity (1.002 mPa^∗^s) and here we have no hosting container but only a sewing thread to hold the phantom in place, the suspended phantom pendulum under water has little friction when compared to the conventional experimental method to characterize calculus migration utilized as a hosting container (e.g., a “V” grove or a test tube) [40] and was almost free to move in the direction perpendicular to the hanging string. Furthermore, a high-speed camera was used to study the movement of the calculus. The SA5 camera from Photron (SA5 16G BW, Photron USA Inc., San Diego, California, USA) is capable of 1 million frames per second (FPS). The retropulsion videos taken with 100,000 fps were analyzed by a MatLab program for the pendulum swing amplitude.

**Figure 4:**
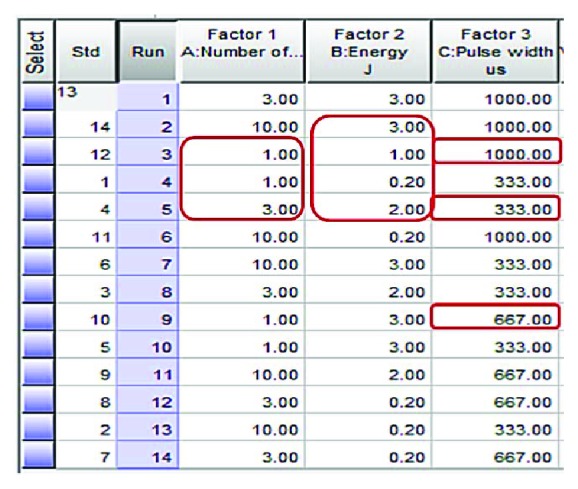
A screen shot of design of experiment by Design-Expert 10.

Figure 4 is a screen shot of design of experiment by Design-Expert 10. The laser parameter settings were listed in three categories: five energy levels (0.2, 0.5, 1, 2, 3 J, and 0.5 J were not selected by the DOE software); three number of pulses (1, 3, and 10); and three electrical pump pulse widths (333, 667, and 1000 *μ*s). The ten pulse range was chosen because typical retropulsion of a 10 × 10 × 10 mm^3^ with 1 J pulse train at 10 Hz will reach its maximum amplitude from the fiber tip after ~1 s [40]. There are 5 × 3 × 3 = 45 data points with the combination of all the laser parameters. As a standard data collection convention, each test was repeated for ten times and each data point is an average of these measurement results. With the help of the Design-Expert 10 software, by using best fit for the surface response, test data runs were reduced to 14 with 12 independent data points (two repeats).

## 3. Results

### 3.1. Retropulsion Amplitude Data

**Figure 5:**
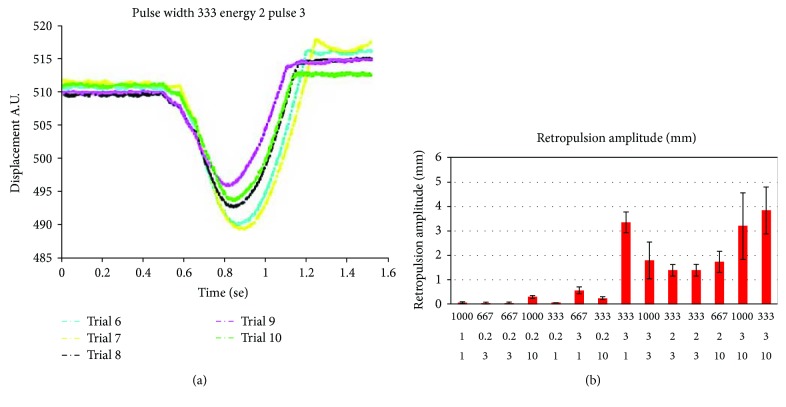
Retropulsion amplitude measurement results. (a) Retropulsion amplitude against time. (b) Retropulsion amplitude response measurement results.

**Figure 6:**
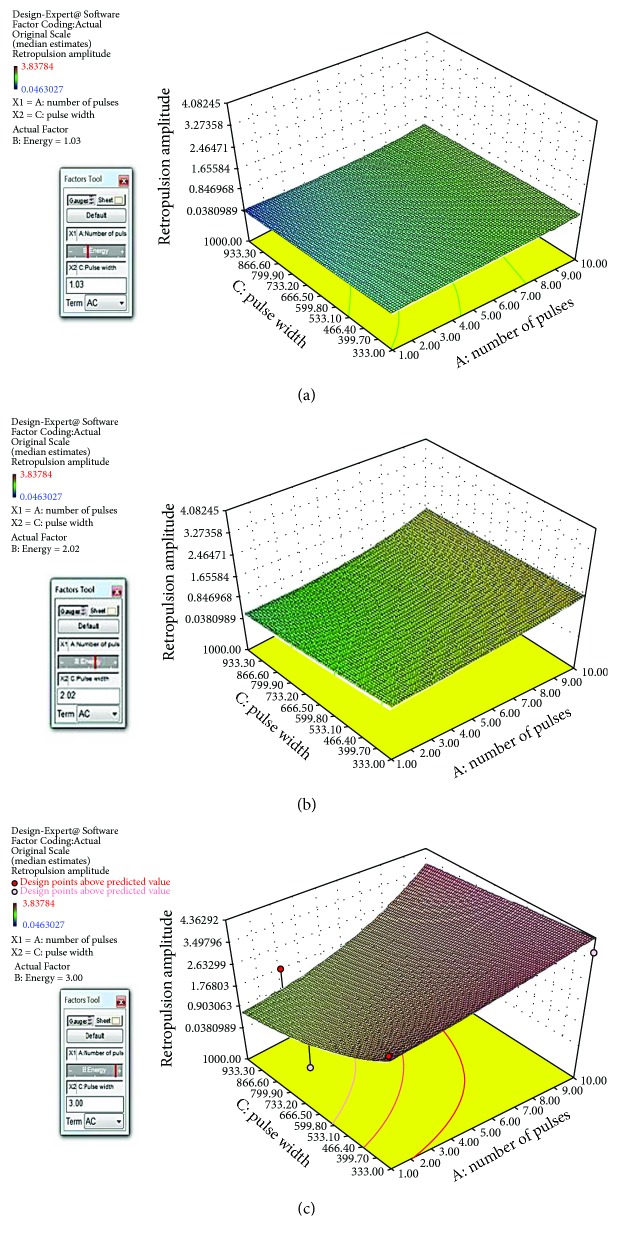
The screen shots of the response surface of retropulsion amplitude against pulse width and number of pulses at pulse energy levels of (a) 1 J, (b) 2 J, and (c) 3 J.

The phantom retropulsion after laser pulse interaction was measured by a high-speed camera (SA5 16G BW, Photron USA Inc., San Diego, California, USA) with a frame rate of 10 kFPS. The retropulsion videos were analyzed by a MatLab program for the pendulum swing amplitude.  Figure 5(a) is some sample curves of the retropulsion movement. The 12 responses of retropulsion amplitude are shown in Figure 6(b); each data point is the average of 10 measurements. The horizontal axis is the laser pulse length, pulse energy, and the number of pulses, while the vertical axis is the retropulsion amplitude in millimeter.

### 3.2. Retropulsion Amplitude Response Surface

Based on the response data from the last section, the Design-Expert 10 software can generate a response surface and the analytical formula of the response surface. There are two responses: one is retropulsion amplitude, and the other is the volume of ablation. The response surface is a 2-dimensional surface of response in terms of the laser pulse setting (pulse length and number of pulses).  Figure 6 is the screen shots of the response surface of retropulsion amplitude in mm against pulse width and number of pulses at pulse energy levels of (a) 1 J, (b) 2 J, and (c) 3 J. The analytical formula of the response surface of retropulsion is shown as follows:

(1) $$\begin{array}{c} A=e^{0.56+0.08n+1.42\varepsilon -0.0021\tau -0.039n\varepsilon +0.00022n\tau -.00011\varepsilon \tau }, \end{array}$$

where *A* is the retropulsion amplitude (mm), *n* is the number of pulses, *ε* is the laser pulse energy (J), and *τ* is the laser pulse width (*μ*s).

### 3.3. Volume of Ablation Data

**Figure 7:**
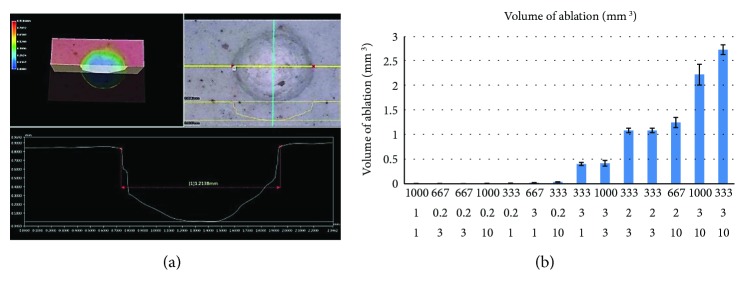
Volume of ablation response measurement results. (a) Screen shot of VHX-900F digital microscope. (b) Volume of ablation response measurement results.

**Figure 8:**
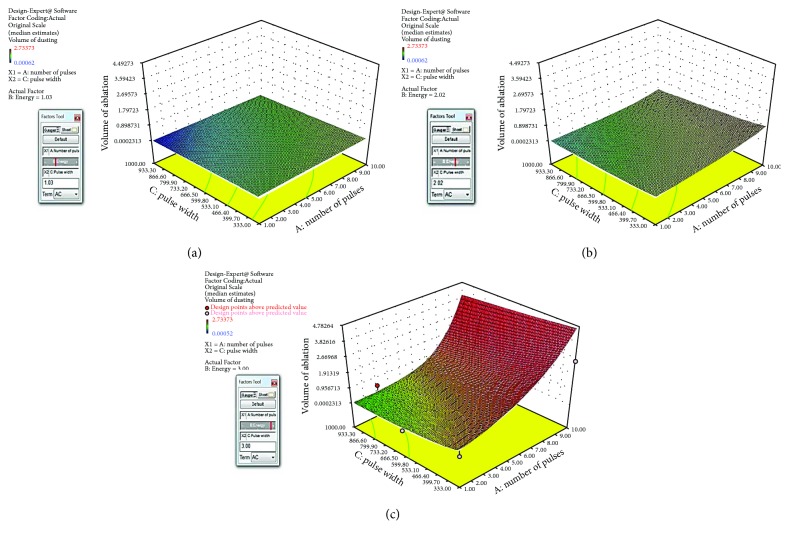
The screen shots of the response surface of volume of ablation with two factor interaction fit against pulse width and number of pulses at pulse energy levels of (a) 1 J, (b) 2 J, and (c) 3 J.

The laser ablation crater volume in the phantom was measured by a digital microscope. A typical image is in Figure 7(a). The 12 responses of volume of ablation are shown in Figure 8(b); each data point is the average of 10 measurements.

### 3.4. Volume of Ablation Response Surface

**Figure 9:**
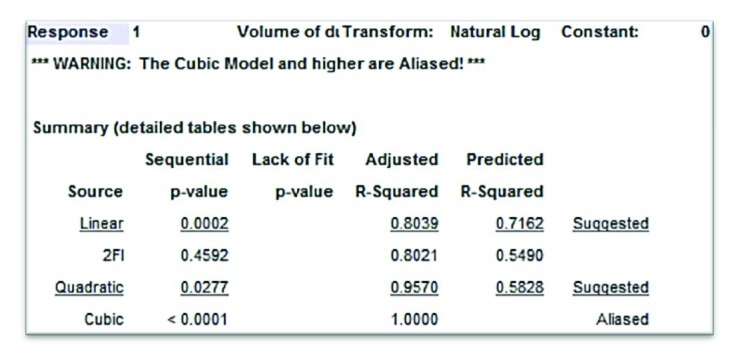
The fit summary (a screen shot) of the response surface of volume of ablation against pulse width, number of pulses, and pulse energy.

Based on the response data from the last section, the Design-Expert 10 software can generate a response surface and the analytical formula of the response surface.  Figure 8 is the screen shots of the response surface of volume of ablation against pulse width and number of pulses at pulse energy levels of (a) 1 J, (b) 2 J, and (c) 3 J. This response surface is under the same assumption of the one for retropulsion in Section 3.2, which includes the polynomial terms of two factor interactions. However, the Design-Expert 10 software suggested that the best fit is in the form of quadratic fit (*p* value of 0.028 and adjusted *R* squared of 0.9570) as shown in Figure 9. The analytical formula of the response surface of the volume of ablation including the polynomial terms of two factor interactions is shown as follows:

(2) $$\begin{array}{c} V=e^{-2.27+0.023n+1.11\varepsilon -0.0083\tau +0.011n\varepsilon +0.00047n\tau +0.0012\varepsilon \tau }, \end{array}$$

where *V* is the volume of ablation (mm^3^), *n* is the number of pulses, *ε* is the laser pulse energy (J), and *τ* is the laser pulse width (*μ*s).

**Figure 10:**
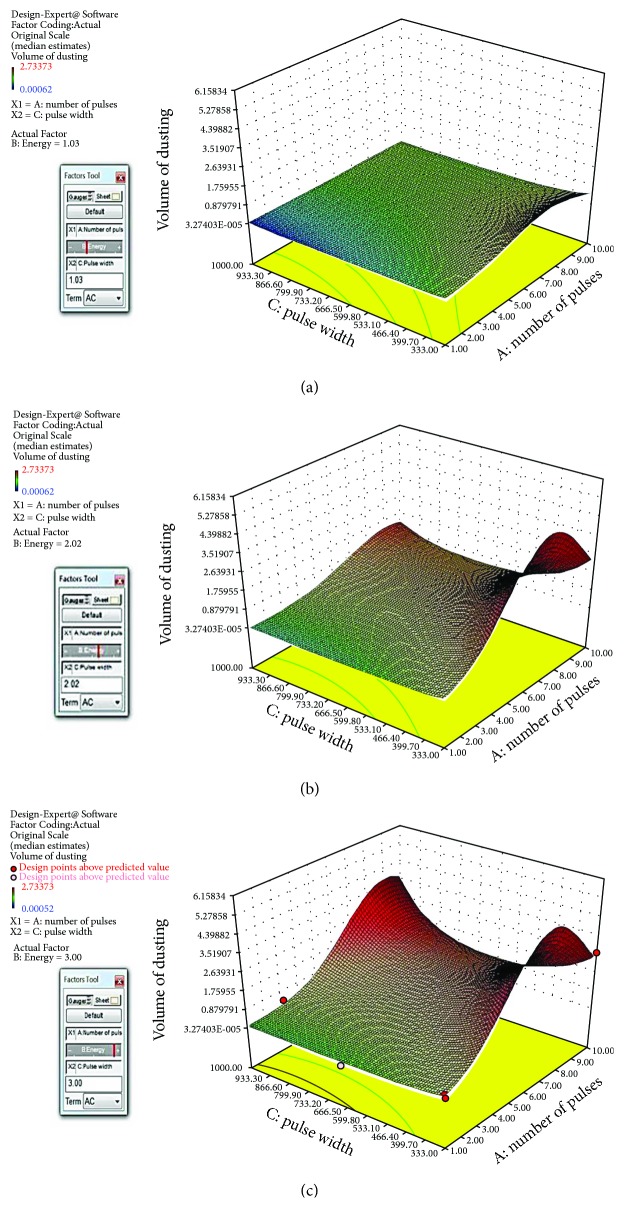
The screen shots of the response surface of volume of ablation with quadratic fit against pulse width and number of pulses at pulse energy levels of (a) 1 J, (b) 2 J, and (c) 3 J.

Figure 10 is the screen shots of the response surface of volume of ablation with quadratic fit against pulse width and number of pulses at pulse energy levels of (a) 1 J, (b) 2 J, and (c) 3 J. The analytical formula of the response surface of volume of ablation including the polynomial terms of two factor interactions is shown as follows:

(3) $$\begin{array}{c} V=e^{-1.16+0.94n+3.46\varepsilon -0.021\tau -0.0031n\varepsilon +0.00048n\tau +0.0014\varepsilon \tau -0.078n^{2}-0.77\varepsilon^{2}+.0000093\tau^{2}}, \end{array}$$

where *V* is the volume of ablation (mm^3^), *n* is the number of pulses, *ε* is the laser pulse energy (J), and *τ* is the laser pulse width (*μ*s).

**Figure 11:**
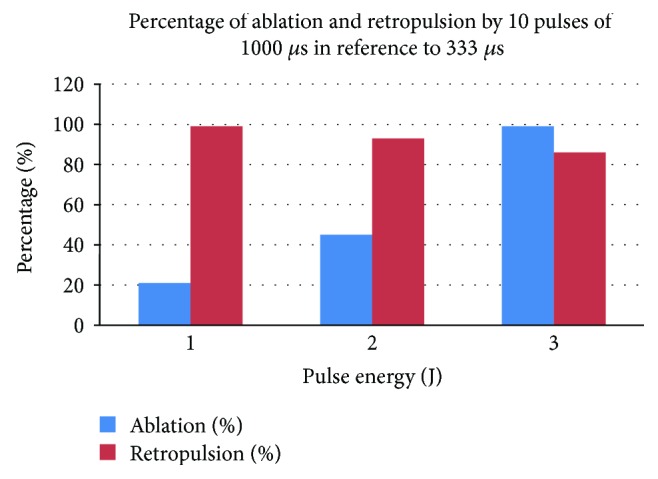
The percentages of ablation and retropulsion by 10 pulses of 1000 *μ*s in reference to those of 333 *μ*s.

Figure 11 is the percentages of ablation and retropulsion by 10 pulses of 1000 *μ*s in reference to those of 333 *μ*s. The difference of volume of ablation between long and short pulses is relatively bigger at 1 J and 2 J levels compared to retropulsion. In other words, ablation decreases faster than retropulsion by increasing pulse length.

## 4. Discussion

Although the rising prevalence of calculus disease in the United States has led to similarly increasing efforts to optimize ureteroscopic treatment [33–40, 46–52], the optimal laser settings for URS for effective treatment remain unclear. In part, this is due to those existing holmium:YAG laser lithotripters allowing operators to control a few laser parameters: pulse energy, pulse width, and pulse frequency. Understanding these adjustable parameters allows the urologist to enhance their control during laser lithotripsy procedures [42]. The aim of this study was to look for systematic quantitative response surfaces of calculus ablation and retropulsion by performing operator-independent, reproducible experiments to determine the best fit of surface responses on volume of ablation and retropulsion amplitude versus the pulse energy, pulse width, and the number of pulses. A design of experiment software (Design-Expert 10, Minneapolis, MN, USA) was used for the surface response methodology (RSM). This can not only cut down the number of test points but also generate a formula for the response surface of ablation speed and retropulsion amplitude. This analytical formula is a useful tool to quantify the response by the laser settings, and it can be used to qualitatively predict even beyond the tested laser settings. In this study, a lab-built Ho:YAG laser was used as the laser pulse source, with a pulse energy from 0.2 J up to 3.0 J and an electrical pump pulse width from 150 *μ*s up to 1000 *μ*s. Based on the 12 tested independent data points, two kinds of response surface formulas were generated for the volume of ablation and retropulsion amplitude, respectively. These polynomial formulas provide a detailed quantitative response of the two key laser calculus interaction effects (volume of ablation and retropulsion) by laser parameters.

By comparing the formulas' polynomial terms up to two factor interactions (2FI), (1) and (2), the dominant factor is the pulse energy with the biggest coefficient, and the pulse energy has more influence on retropulsion than ablation (1.42 versus 1.11). And for the pulse width effect, the longer the pulse, the less the ablation or retropulsion, while the longer pulse is less efficient for ablation of the stone, compared to the effect on retropulsion (−0.0083 versus −0.0021), as shown in Figure 11. Overall, the two factor terms have a few times to an order of magnitude less influence compared to the first order terms.

**Figure 12:**
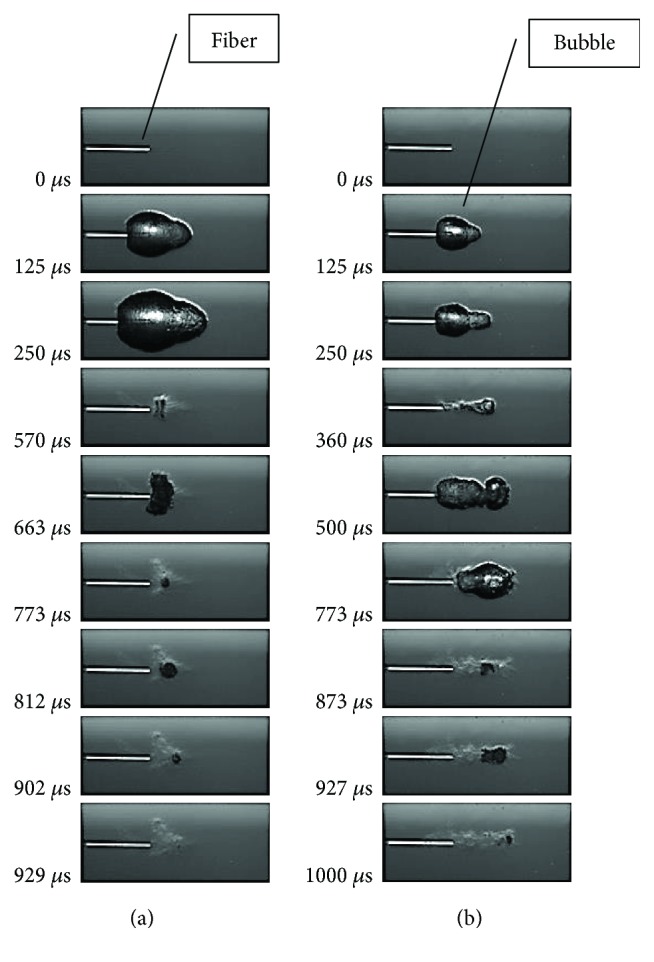
Series of screen shots of cavitation bubble behavior of Ho and Tm lasers. (a) Ho at 1 J, 150 *μ*s; (b) Ho at 1 J, 800 *μ*s.

Figure 10 and (3) are the best quadratic fit for the volume of ablation which has evidently nonlinear effects between long and short pulses at pulse number ~7-8. That is when pulse number is ~7-8, the volume of ablation has a saddle shape along the pulse width axis. This can be explained by the cavitation bubble dynamics study in [46]. As it is shown in Figure 12(b) [46], the cavitation bubble of long laser pulse will have a much elongated bubble which is composed of two small bubbles with sequential collapse times, and the 2nd bubble collapses further away from the fiber tip as compared to the short laser pulse case in (a). Therefore, the long laser pulse can reach and interact further away from the fiber tip and make deeper crater or bigger volume of ablation. This effect is stronger at a higher pulse energy as shown in Figure 10(c), and the depth of the hole has a limit which ends ~7-8 pulses because both fiber and calculus were fixed.

We wanted to note that the range of testing conditions in this study are 0.2–3 J, 333–1000 *μ*s, and 1–10 pulses (10 Hz); the calculus phantom is gypsum white cement, the phantom is fixed in a holder, and only 356 *μ*m core diameter fiber is used for testing. There is another well-known issue in laser lithotripter: fiber tip burn back [44, 53, 54], which is also a key factor for procedure time, patient safety, and care economics. Further study should explore laser settings beyond the current range, and fiber burn back should be taken into account when searching for the optimum laser setting for urolithiasis. More detailed investigation of the optimal conditions for the ablation of other kinds of calculus samples [55], actual human calculus, and the fiber size effect will also be conducted as a future study.
